# Immune evasion mechanisms of arenaviruses

**DOI:** 10.18632/oncotarget.6367

**Published:** 2015-11-22

**Authors:** Hinh Ly, Yuying Liang

**Affiliations:** Department of Veterinary & Biomedical Sciences, University of Minnesota, Twin Cities, MN, USA

**Keywords:** arenavirus, immune evasion, lassa virus

Arenaviruses, such as Lassa virus (LASV), can cause fatal human hemorrhagic fever (HF) diseases for which licensed vaccines and effective therapies are currently unavailable [1]. These viruses have been shown to initiate the infection by targeting the antigen presenting cells (APCs), such as dendritic (DC) and macrophage (MP) cells [1]. While MPs and DCs are readily infected with arenaviruses (e.g., LASV), they fail to become activated upon infection, the molecular mechanisms for which are unclear. LASV-infected APCs show no increases in the levels of activating markers, such as CD80, CD86, CD40, CD54, HLAs, and of cytokines, such as TNFα, IL-1β, IL-6, IL10 or IL-12 [2, 3]. Also, LASV-infected DCs fail to mature as evidenced by the failure to up-regulate costimulatory molecules, and as a result, they are poor stimulator of T cells in culture [3]. They also show poor levels of phagocytic activity [2]. The failure of APCs to become activated upon viral infection is consistent with the generalized immune suppression that is one of the known hallmarks of LASV infection.

We have recently shown for the first time that the small (11 kD) Z proteins from all known pathogenic arenaviruses (including LASV), but not those of the non-pathogenic arenaviruses, can inhibit type-I IFNs by directly binding to the cytosolic innate-immune sensor proteins RIG-I and MDA-5 and that these interactions interfere with the binding of these proteins to a downstream signaling protein MAVS, thus preventing the production of type-I IFNs [4]. We also show that this is dependent only on the N-terminal domain of the pathogenic arenaviral Z proteins. In a follow-up study [5], we show that primary human MPs, upon infection with pathogenic arenaviruses, fail to elevate the levels of activating markers, such as CD80, CD86 and of cytokines, such as IFN-β, TNFα, IL-1β, IL-1β, IL-6, and IL-8. Also, infected human primary MPs fail to mature, as evidenced by the absence of increased levels of phagocytic activity. The failure of these APCs to become activated upon pathogenic viral infection in turn leads to the inability to mount effective T-cell and NK-cell responses as evidenced by the lack of IFN-γ production in the MP-T cells and MP-NK co-cultures. The results of our recent studies [4, 5] indicate that the pathogenic Z-mediated type-I IFNs inhibition may be a common pathogenic mechanism underlying the diverse arenavirus pathogens. While there are potentially several factors that can lead to highly variable disease symptoms in humans infected with arenaviruses, all of the known pathogenic viruses encode a Z protein that can inhibit the type-I IFNs production in cell culture. We therefore propose that the innate immune suppressive effect of the Z protein may help establish the potential for certain arenaviruses to cause human diseases. Whether this potential leads to a self-limiting infection or severe diseases depends on multiple other factors such as the virus strains, viral inoculum dose, infection route, host immune status, pre-existing immunity, and genetic variations. Further studies are therefore necessary to fully understand the relationship among these virulent factors and/or mechanisms leading to clinical diseases in patients infected by different arenavirus pathogens. Our recent studies [4, 5] collectively implicate for the first time an important role of the viral factor (Z) in defining pathogenic arenaviral infections, thereby revealing a new and specific target for potential development of antiviral drugs against deadly hemorrhagic fever causing arenaviruses.

Like LASV, infections by other so-called Old World pathogenic arenaviruses are characterized by the inability of the infected cells to mount an effective innate immune response [6]. We and other researchers have demonstrated that the viral nucleoprotein (NP) serves as a potent inhibitor of type-I IFNs production by degrading immune stimulatory RNAs [7] or through other mechanisms [reviewed in [6]]. After extensive investigations, it appears that the NP-mediated type I IFN suppression is a general mechanism of immune suppression mediated by both pathogenic and non-pathogenic arenaviruses. The next and obvious question is how these two viral IFN antagonists (Z and NP) function in immune cells (APCs) to evade host recognition in order to inhibit type-I IFNs production and/or signaling.
